# Evaluation of a prognostic model for risk of relapse in stage I seminoma surveillance

**DOI:** 10.1002/cam4.324

**Published:** 2014-09-19

**Authors:** Peter Chung, Gedske Daugaard, Scott Tyldesley, Eshetu G Atenafu, Tony Panzarella, Christian Kollmannsberger, Padraig Warde

**Affiliations:** 1 Radiation Medicine Program, Princess Margaret Cancer CentreToronto, Canada; 2 Department of Radiation Oncology, University of TorontoToronto, Canada; 3 Department of Oncology, RigshospitaletCopenhagen, Denmark; 4 British Columbia Cancer AgencyVancouver, Canada; 5 Department of Biostatistics, Princess Margaret Cancer CentreToronto, Canada

**Keywords:** Prognostic model, stage I seminoma, surveillance

## Abstract

A prognostic model for relapse risk in stage I seminoma managed by surveillance after orchiectomy has been developed but has not been independently validated. Individual data on 685 stage I seminoma surveillance patients managed between 1998 and 2005 at three cancer centers were retrospectively analyzed. Variables including age and pathology of the primary tumor: small vessel invasion, tumor size, and invasion of rete testis were analyzed. Specifically median tumor size and rete testis invasion was tested to evaluate the performance of the published model. Median follow-up was 3.85 years (0.1–10.29), 88 patients relapsed and 5-year relapse-free rate was 85%. In univariate analysis, median tumor size (<3 cm vs. ≥3 cm) was associated with increased risk of relapse but rete testis invasion was not, nor was age and small vessel invasion. In multivariable analysis, tumor size above median (cutpoint of 3 cm) was a predictor for relapse, HR 1.87 (95% CI 1.15, 3.06), whereas rete testis invasion HR 1.36, (95% CI 0.81, 2.28) was not statistically significant. The 3-year relapse risk based on the primary tumor size alone increased from 9% for 1 cm primary tumor to 26% for 8 cm tumor. A clinically useful, highly discriminating prognostic model remains elusive in stage I seminoma surveillance as we were unable to validate the previously developed model. However, primary tumor size retained prognostic importance and a scale of relapse risk based on the unit increment of tumor size was developed to help guide patients and clinicians in decision making.

## Introduction

Historically adjuvant radiotherapy (RT) has been the most popular treatment option after radical orchiectomy for stage I testicular seminoma 1; however, better understanding of the natural history of the disease, the advent of highly successful combination chemotherapy in patients with metastatic disease, and accumulating long-term data suggesting potentially serious consequences of adjuvant RT 2–4 lead to the investigation of alternative strategies, thus significantly altering management options 5–8. Although adjuvant therapy (RT or carboplatin) remains an option for some patients, surveillance is now well established as the preferred management option for most patients in this setting. Only those men who develop relapse will receive additional treatment, while all others, who are cured of their disease by orchiectomy alone, will not be exposed to the risk of adverse effects associated with adjuvant therapy. However, there are some issues with respect to surveillance that have resulted in reluctance to adopt this approach in stage I seminoma 9,10. These include the need for a relatively long period of follow-up, with regular cross sectional imaging and the lack of a robust prognostic model to identify those men at higher risk of relapse.

A prognostic model derived from multi-institutional data to estimate risk of relapse in stage I seminoma patients managed with surveillance was published in 2002 11. This model was based on two pathological factors in the primary tumor specimen, tumor size and the presence or absence of rete testis invasion. This model has been adopted by some groups to investigate “risk-adapted” therapy 12 despite the fact that the model did not have a very useful discriminatory ability. This approach is still considered as experimental in consensus guidelines 13,14. In addition, the model has never been subject to full validation in an independent dataset. The purpose of this study was to assess the performance of the 2002 prognostic model in an independent dataset of patients managed with surveillance for stage I seminoma.

## Patients and Methods

### Patient population and data collection

After research ethics board approval, individual data on 685 patients were obtained from 3 centers: Rigshospitalet, Copenhagen; Princess Margaret Cancer Centre, Toronto; British Columbia Cancer Agency, Vancouver. The data were contained in prospectively managed databases at the three institutions. All patients had orchiectomy and negative staging investigations which included chest X-ray and/or computed tomography (CT) scan of the thorax, CT scan of the abdomen and pelvis (CTAP), and serum tumor markers; alpha fetoprotein, human chorionic gonadotropin, lactate dehydrogenase (HCG, AFP, LDH), to confirm clinical stage I disease. All stage I seminoma patients managed by surveillance at participating centers between 1998 and 2005, were included in the analysis. The choice of surveillance as a management strategy was independent to this study, and patients were placed on the surveillance schedule specific to each center. The following data were collected: age at diagnosis, date of diagnosis, date of last follow-up, relapse status, date of relapse (if any), and survival status at last follow-up, as well as histologic features and size of the primary tumor. While central pathology review was not conducted the diagnosis had been confirmed by experienced pathologists.

At relapse, patients were managed according to local policies, which usually included either para-aortic and pelvic RT or combination cisplatin-based chemotherapy, depending on the local preference and the extent of disease at relapse.

### Statistical analysis

Categorical variables such as presence and absence of rete testis invasion, and of small vessels, that is, lymphovascular invasion, were summarized with counts and percentages. Continuous variables such as age at surgery, tumor size, and follow-up were summarized with mean and standard deviation or medians and/or ranges as necessary.

The primary outcome variable was time to relapse and was calculated as the time in years from the date of orchidectomy to the date of relapse for those who relapsed and to last date of follow-up for those who did not. The Kaplan–Meier product method was used to generate relapse-free survival (RFS) curves and estimated RFS probabilities. Univariate analyses utilized log-rank testing, and multivariable analyses incorporated the Cox Proportional Hazards model. SAS version 9.2 (SAS Institute Inc., Cary, NC) was used to perform all statistical analyses. Two-sided *P* < 0.05 was used to determine statistical significance.

## Results

**Figure 1:**
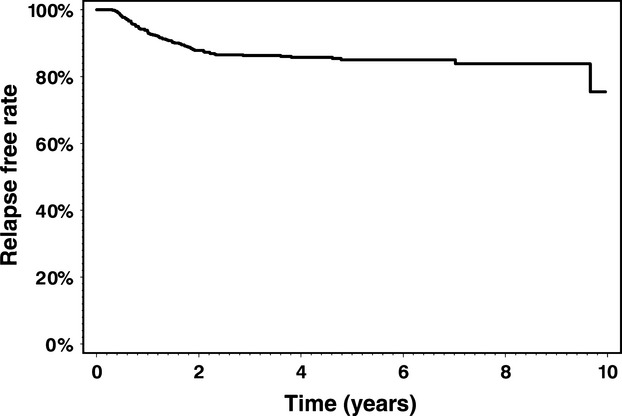
Relapse-free rate amongst 685 patients with stage I seminoma managed with surveillance after orchiectomy.

The three centers identified a total of 685 stage I seminoma patients managed with surveillance. Patient characteristics are shown in Table1. One patient was excluded from the analysis due to lack of follow-up data. The median age of patients was 36 years (range = 16–82) and the median tumor size was 3 cm (range = 0.2–13). With a median follow-up of 3.85 years (range = 0.1–10.29), 88 patients had developed relapse. The actuarial relapse-free rate at 3 and 5 years was 86.3% and 85%, respectively (Fig.1). Median time to relapse was 12 months (range = 3.7–116 months) and 84% (*n* = 74) of the relapses occurred within the first 2 years of follow-up. At time of last follow-up, six patients had died, none of whom had disease-related death.

**Table 1:** Patient characteristics.

Variable	Category	All (685)
Tumor size	≤4 cm	408
	>4 cm	161
	Missing	116 (16.9%)
Rete testis invasion	Absent	312
	Present	166
	Missing	207 (30.2%)
Age at surgery	≤36	361
	>36	323
	Missing	1 (0.15%)
Small vessels invasion	Absent	462
	Present	50
	Missing	173 (25.3%)

### Prognostic factors for relapse

The candidate prognostic factors tested included age at diagnosis, primary tumor size, rete testis invasion, and small vessel invasion. On univariate analysis none of these factors apart from primary tumor size were statistically associated with an increase in relapse risk (Table2). Larger tumors were statistically associated with an increased relapse risk when analyzed as a dichotomous variable, using median tumor size, 3 cm, as a cutpoint (*P* = 0.01), or as a continuous variable (*P* = 0.0006). On multivariable analysis, patients with primary tumor size ≥3 cm had 1.87 times higher risk of relapse, (95% confidence interval [CI] 1.15–3.06, *P* = 0.01) compared to those with tumors less than 3 cm. Patients with rete testis invasion did not have a statistically significant elevated risk of recurrence, HR 1.36 (95% CI 0.81–2.28, *P* = 0.25).

**Table 2:** Univariate prognostic factor analysis.

Variable	Category	2 year RFR	3 year RFR	*P*-value
Tumor size*	≤4 cm	0.8972	0.8779	0.0473
	>4 cm	0.8168	0.8088	
Tumor size	<3 cm	0.9216	0.9064	0.0121
	≥3 cm	0.8326	0.8158	
Tumor size	Continuous	0.8785	0.8631	0.0006
Rete testis	Present	0.8321	0.8156	0.2319
	Absent	0.8855	0.8651	
Small vessel invasion	Absent	0.8704	0.8506	0.5242
	Present	0.9032	0.8731	
Age	≤36	0.8958	0.8574	0.2749
	>36	0.8593	0.8411	
Age	Continuous	0.8785	0.8631	0.7746

* Cutpoint from previously published analysis 11.

### Relapse risk based on primary tumor size alone

The risk of relapse observed increased from 2% at 3 years for tumor size 1 cm or less to 25% for tumor size of 6 cm or greater. From this we developed a model to estimate 3-year relapse rates for patients relative to the unit increment of the primary tumor size. The estimate for a patient with primary tumor size of 1 cm was 9% and this increased to 26% for a patient with a tumor size of 8 cm (Table3).

**Table 3:** Primary tumor size and predicted 3 year relapse risk for stage I seminoma surveillance patients.

Primary tumor size*	Rate of relapse (%)
1 cm	9
2 cm	11
3 cm	13
4 cm	15
5 cm	17
6 cm	20
7 cm	23
8 cm	26

* Insufficient data to estimate rates for tumor size greater than 8 cm.

## Discussion

In this study, the performance of the previously published prognostic model for risk of relapse in stage I seminoma patients managed by surveillance was poor and had limited ability to predict relapse. We found insufficient evidence to support the findings of the previous study where the prognostic model, based on the two factors, presence of primary tumor size of ≥4 cm (median cutpoint) and rete testis invasion, indicated an increased risk of relapse of 35%. As such, the use of this model for treatment decision making in stage I seminoma patients to determine adjuvant therapy or surveillance cannot be routinely recommended. Despite this, we were able to confirm that primary tumor size alone retains prognostic importance for the estimation of recurrence risk after orchiectomy alone. Men with primary tumor size greater than the median cutpoint (3 cm) were overall 1.87 times more likely to relapse than those with less than <3 cm tumor. This translates into 18.4% risk of relapse at 3 years for a patient with ≥3 cm tumor and if this directed the use of adjuvant therapy would still require four out of every five men to be thus unnecessarily treated. As median tumor size may have limited clinical application when discussing an individual patient's relapse risk, we sought to further illustrate this by defining the predicted relapse risk for a given primary tumor size. A small but steady increase in relapse risk with unit increase in primary tumor size was seen in the current cohort and there was a consistent effect of tumor size seen when compared to the previous dataset in addition when using the previous model with both factors the predicted relapse would be expected to be 10% (P. Warde, pers. comm.). This information may thus be useful when counseling patients about their specific relapse risk based on the primary tumor size. However given that the highest predicted risk for tumor size of 8 cm was still under 30%, we would not routinely recommend adjuvant therapy, as such patients would have over 70% chance of never requiring further therapy after radical orchiectomy.

Since the publication of the prognostic model in 2002, two studies by the Spanish Germ Cell Cancer Cooperative group have been undertaken. The first included patients who were considered to be at high risk of recurrence based on the published model, managing such patients with adjuvant carboplatin chemotherapy 12. Patients with either or both risk factors were given two cycles of carboplatin (AUC-7), while all other patients were managed by surveillance. Prognostic factors for relapse were analyzed, and for patients who received carboplatin, rete testis invasion was a statistically significant predictor for relapse (*P *= 0.01) despite this adjuvant therapy. It remains unclear as to the clinical utility of this observation given that all who had rete testis patients invasion had adjuvant therapy and might not be expected to still have an increased relapse risk. The same authors subsequently conducted a similar study with 227 patients but restricted adjuvant chemotherapy to those who had both risk factors present 15. The results of this study showed excellent relapse-free rates, as expected, for those treated with adjuvant carboplatin but interestingly the relapse rate for surveillance patients with rete testis invasion alone (*n* = 25) was 20%, although a small number, this was similar to that of an unselected population which would be expected to be 15–20% in most reports of surveillance. Although both these studies might be viewed as partial validation of the prognostic model (to distinguish a group at low risk of relapse), overall the discrimination between low-and high-risk groups is not powerful enough to direct adjuvant treatment, as in the majority of cases in the high-risk group would still receive unnecessary treatment.

Rete testis invasion was found to be a predictive factor for recurrence in a Japanese study^16^. A retrospective analysis of 425 patients managed with either surveillance or adjuvant therapy (RT or chemotherapy) between 1985 and 2006 indicated that rete testis invasion was an independent predictive factor for relapse (HR: 5.83, 95% CI 1.83–18.6, *P* = 0.003). However, analysis of the surveillance cohort alone did not support the predictive nature of rete testis involvement. In addition, the authors reported that more than 56% of patients were lost to follow up. Given the aforementioned issues, these data should be viewed with some caution.

A population-based study from Scandinavia 17 with data prospectively collected, examined 1384 stages I and II seminoma patients managed with various strategies. Of these 512 patients stage I patients were managed by surveillance and 14.3% (*n* = 65) relapsed. No relationship between relapse for patients managed by surveillance and vascular invasion (*P* = 0.103), tumor markers (*P* = 0.102), age (*P* = 0.462), or tumor size (*P* = 0.186) was found and it was concluded that clinical factors alone were not sufficient for a risk-adapted management in stage I seminoma. Unfortunately, rete testis invasion was not recorded in that study, although the authors suggested that as the time period in their study was when the data with respect to rete testis as a risk factor became available, based on the published model 11 and clinical practice guidelines 18, physicians may have been influenced in the selection of treatment based on this factor. Similarly primary tumor size may have also played a role in the selection of management in this patient cohort. As there was no randomization between treatment groups, there may possibly have been bias in the selection of patient groups that were allocated for adjuvant treatment and for surveillance.

Limitations of this study include its retrospective nature, the proportion of missing data, the lack of central pathology and imaging review to ensure consistency of definition of disease state. Selection bias is always an issue that may affect the results of any nonrandomized study. While all centers that contributed data to the current study support surveillance as the preferred management option for all stage I seminoma patients without consideration of risk stratification, the overall influence of the prognostic model on the choice of surveillance versus adjuvant therapy for individual clinicians and patients is not known. Although these data were prospectively collected within each of the participating center's database, the analysis is limited by its retrospective nature. When comparing the case mix between the current cohort and the previously published cohort, the current cohort had smaller tumors (which could be as a result of selection bias in our cohort or due to patients having smaller tumors at presentation), there were also more missing data for tumor size (17% vs. 6.3%) and small vessel invasion (25% vs. 9.9%). The median follow-up in the current cohort was shorter than the previous cohort (7 years) resulting in a lower overall proportion of relapses in the present study (12.8% vs. 19%).We acknowledge that some of these differences may have effects on the present study, in particular similar to the Scandinavian study, there may have been an element of selection for adjuvant therapy in the individual centers. Unfortunately we were unable to obtain all the corresponding data for patients that had been managed with adjuvant therapy in this time-frame from the centers. However, the majority of patients at each center was preferentially managed with surveillance and adjuvant therapy was generally used only when this was the patient's preference and not based on any other features. At Princess Margaret, for example, less than 5% of patients with stage I seminoma are managed with adjuvant therapy. The issue of shorter follow-up time in this study and the lower relapse rate is ameliorated by the fact that the majority of patients with stage I seminoma relapse within the first 2–3 years in most studies and the relatively smaller proportion of patients who relapse later may not substantially alter the outcome of this analysis.

Although the current presented data may allow for an informed discussion with respect to selection of adjuvant therapy versus observation in this disease, treatment-associated toxicity (regardless of the timing of that treatment) becomes a much more important element in the management decision process after orchiectomy. It is clear that further effort is required in order to better identify patients who have micrometastatic disease after orchiectomy and thus will require treatment. This is likely to be in the form of molecular or tumor genetic factors that are yet to be determined. Recent work has used whole genome sequencing of the primary tumor in a limited number of patients with metastatic seminoma 19. Specific genes that were found to have a significant association with metastatic seminoma were dopamine receptor D1 (DRD1) and family with sequence similarity 71 (FAM71F2), and when combined into a single model had 87% concordance. In addition small RNA copy number changes may be able to even better discriminate between metastatic and nonmetastatic disease 20. We are currently embarking on such a study in a smaller cohort of patients who have available tumor specimens for testing. If such findings are confirmed on subsequent studies, this may pave the way for more individualized decision making for patients with stage I seminoma.

## Conclusions

While tumor size retained prognostic importance, the previously developed prognostic model for relapse was not validated. We were able to develop an estimate of relapse risk based on unit increment in primary tumor size that may be of some value when counseling patients as to their specific relapse risk when pursuing a surveillance strategy. The use of risk-adapted therapy based on this model is not recommended and a clinically useful prognostic model for stage I seminoma patients to direct management after orchiectomy remains elusive.

## Conflict of Interest

None declared.

## References

[b1] Ramakrishnan S, Champion AE, Dorreen MS, Fox M (1992). Stage I seminoma of the testis: is post-orchidectomy surveillance a safe alternative to routine postoperative radiotherapy?. Clin. Oncol. (R. Coll. Radiol.).

[b2] Travis LB, Curtis RE, Storm H, Hall P, Holowaty E, Van Leeuwen FE (1997). Risk of second malignant neoplasms among long-term survivors of testicular cancer.[see comment]. J. Natl. Cancer Inst.

[b3] Travis LB, Fosså SD, Schonfeld SJ, McMaster ML, Lynch CF, Storm H (2005). Second cancers among 40,576 testicular cancer patients: focus on long-term survivors. J. Natl. Cancer Inst.

[b4] Fosså SD, Travis LB, Bokemeyer C, Dahl AA, Laguna MP, Albers P, Richie JP (2011). Testicular cancer: late effects of treatment. Cancer of the testis.

[b5] Chung P, Mayhew LA, Warde P, Winquist E, Lukka H (2010). Management of stage I seminomatous testicular cancer: a systematic review. Clin. Oncol. (R. Coll. Radiol.).

[b6] Chung P, Warde P (2011). Stage I seminoma: adjuvant treatment is effective but is it necessary?. J. Natl. Cancer Inst.

[b7] Horwich A, Huddart R (2010). Testicular seminoma: ESMO Clinical Practice Guidelines for diagnosis, treatment and follow-up. Ann. Oncol.

[b8] Kollmannsberger C, Tyldesley S, Moore C, Chi KN, Murray N, Daneshmand S (2011). Evolution in management of testicular seminoma: population-based outcomes with selective utilization of active therapies. Ann. Oncol.

[b9] Arvold ND, Catalano PJ, Sweeney CJ, Hoffman KE, Nguyen PL, Balboni TA (2012). Barriers to the implementation of surveillance for stage I testicular seminoma. Int. J. Radiat. Oncol. Biol. Phys.

[b10] Vossen CY, Horwich A, Daugaard G, Poppel H, Osanto S, van (2012). Patterns of care in the management of seminoma stage I: results from a European survey. BJU Int.

[b11] Warde P, Specht L, Horwich A, Oliver T, Panzarella T, Gospodarowicz M (2002). Prognostic factors for relapse in stage I seminoma managed by surveillance: a pooled analysis. J. Clin. Oncol.

[b12] Aparicio J, Germà JR, García del Muro X, Maroto P, Arranz JA, Sáenz A (2005). Risk-adapted management for patients with clinical stage I seminoma: the Second Spanish Germ Cell Cancer Cooperative Group study. J. Clin. Oncol.

[b13] Schmoll HJ, Jordan K, Huddart R, Pes MP, Horwich A, Fizazi K (2010). Testicular seminoma: ESMO Clinical Practice Guidelines for diagnosis, treatment and follow-up. Ann. Oncol.

[b14] Beyer J, Albers P, Altena R, Aparicio J, Bokemeyer C, Busch J (2013). Maintaining success, reducing treatment burden, focusing on survivorship: highlights from the third European consensus conference on diagnosis and treatment of germ-cell cancer. Ann. Oncol.

[b15] Aparicio J, Maroto P, del Muro XG, Gumà J, Sánchez-Muñoz A, Margelí M (2011). Risk-adapted treatment in clinical stage I testicular seminoma: the third Spanish Germ Cell Cancer Group study. J. Clin. Oncol.

[b16] Kamba T, Kamoto T, Okubo K, Teramukai S, Kakehi Y, Matsuda T (2010). Outcome of different post-orchiectomy management for stage I seminoma: Japanese multi-institutional study including 425 patients. Int. J. Urol.

[b17] Tandstad T, Smaaland R, Solberg A, Bremnes RM, Langberg CW, Laurell A (2011). Management of seminomatous testicular cancer: a binational prospective population-based study from the Swedish norwegian testicular cancer study group. J. Clin. Oncol.

[b18] Schmoll HJ, Souchon R, Krege S, Albers P, Beyer J, Kollmannsberger C (2004). European consensus on diagnosis and treatment of germ cell cancer: a report of the European Germ Cell Cancer Consensus Group (EGCCCG). Ann. Oncol.

[b19] Ruf CG, Linbecker M, Port M, Riecke A, Schmelz HU, Wagner W (2012). Predicting metastasized seminoma using gene expression. BJU Int.

[b20] Ruf CG, Schmelz HU, Port M, Wagner W, Matthies C, Müller-Myhsok B (2014). Discriminating metastasised from non-metastasised seminoma based on transcriptional changes in primary tumours using NGS. Br. J. Cancer.

